# Drug resistance mutations among people living with HIV and ART failure in Bangladesh: a cross-sectional study

**DOI:** 10.1016/j.lansea.2025.100629

**Published:** 2025-07-04

**Authors:** Sezanur Rahman, Md Safiullah Sarker, Md Mobarok Hossain, Md Abir Hossain, Mohammad Fakhruddin, Rubel Howlader, Golam Sarwar, Sharful Islam Khan, Mustafizur Rahman

**Affiliations:** a Virology Laboratory, Infectious Diseases Division, icddr,b, Mohakhali, Dhaka, 1212, Bangladesh; b Genome Centre, Infectious Diseases Division, icddr,b, Mohakhali, Dhaka, 1212, Bangladesh; c Programme for HIV and AIDS, Health Systems and Population Studies Division, icddr,b, Mohakhali, Dhaka, 1212, Bangladesh

**Keywords:** Anti-retroviral therapy (ART), Viral load (VL), Drug resistance, Men who have sex with men, Transgender women

## Abstract

**Background:**

While maintaining a low HIV prevalence among the general population, Bangladesh is among the few countries in the Asia–Pacific region where the incidence of people living with HIV (PLWH) continues to increase. The National Anti-Retroviral Therapy Program still relies on a ‘Test and Treat’ strategy and faces challenges in treating drug-resistant HIV. This study aims to assess the HIV viral load and drug resistance mutations among key populations (KPs) under anti-retroviral therapy (ART) in Bangladesh.

**Methods:**

A total of 110 KPs from 20 drop-in-centers across 11 districts in Bangladesh were enrolled from March 2019 to November 2020 for viral load (VL) testing using Xpert® HIV-1 Viral Load kits. Samples with high VL (≥1000 copies/mL) underwent pol gene sequencing to identify drug resistance mutations.

**Findings:**

Most of the participants were men who have sex with men (MSM, 49.1%) followed by men sex workers (MSW, 34.5%), and transgender women (TGW, 13.6%). The median age of the participants was 28 years (IQR: 24–35) and 80% of them were from the capital city, Dhaka. The median time for ART was 11.5 months (IQR: 4.5–29.1), where 15 participants were naïve to ART. Overall, high VL was observed in 23.8%, and virologic failure was in 17.9% among PLWH who were treated for >30 days. HIV-1 subtype C was predominant (43.8%), followed by A1 (25%), CRF01_AE (25%), and CRF02_AG (6.2%). Seven participants showed resistance against Efavirenz, the common drug received from ART centres, and three of them were additionally resistant against Tenofovir Disoproxil Fumarate. Other drugs supplied by ART centres were also found resistant for participants; i.e. 6 against Emtricitabine, 6 against Lamivudine, and 1 against Etravirine. The spatial distribution indicated HIV transmission occurred within and between KPs and drop-in-centers. Additionally, samples that received different ART also clustered together.

**Interpretation:**

Evidence suggests that KPs are at a higher risk of virologic failure in Bangladesh, emphasizing the need for routine VL and drug resistance mutation tests as part of the national ART program. This study also advocates for exploring barriers to ART adherence and implementing personalized ART strategies in national ART programs.

**Funding:**

The Global Fund.

**Research in context**

**Evidence before this study**

On September 3, 2024, we conducted a PubMed search to identify studies reporting HIV viral load (VL) or drug-resistant mutations (DRMs) from Bangladesh (search keywords for Title/Abstract ((‘Dhaka’ OR ‘Bangladesh’) AND ‘HIV’) AND (‘Viral load’ OR ‘VL’ OR ‘DRM’ OR ‘Resistance’)). A total of five articles were identified. One study highlighted barriers that negatively impacted ART adherence^1^ while another reported on chemokine receptor profiles among Bangladeshi people living with HIV.^2^ One study examined the response to first-line ART by assessing CD4, CD8 T-lymphocytes, and VL, but did not report on DRMs.^3^ The remaining two studies identified the presence of DRMs, but VL data were not provided.^4^^,^^5^

**Added value of this study**

This study is among the first to provide detailed data on DRM and VL among key populations (KPs), contributing significant value to HIV research and supporting reforms in the National AIDS/STD Programme (ASP) in Bangladesh. It also offers new insights into HIV transmission within and between KPs by identifying specific clusters and geographic hotspots, offering a snapshot of how the virus spreads in these communities. Furthermore, the study highlights critical gaps in current ART practices, particularly the absence of routine DRM testing and the reliance on a ‘test and treat’ strategy without sufficient VL follow-up. Finally, it delivers actionable recommendations for improving ART practices and HIV management in the country, advocating for the integration of routine VL and DRM testing. Although pre-ART resistance screening is not necessary for the TDF+3TC/FTC + DTG first-line regimen, we recommend integrating a DRM monitoring system alongside VL testing, particularly in resource-limited countries like Bangladesh, where the availability of this regimen may be inconsistent or interrupted. Finally, this study provides concrete evidence of the need for such integration, emphasizing the risks associated with insufficient monitoring.

**Implications of all the available evidence**

The findings provide a foundation for future research and policy development, offering a benchmark for tracking the evolution of drug resistance in Bangladesh. In addition, the study provides actionable, evidence-based policy recommendations for updating national ART guidelines in the country. It specifically advocates for the implementation of drug resistance testing before initiating ART and regular monitoring thereafter, practices that are not yet standard in the country. The study's recommendations have the potential to significantly enhance the effectiveness of ART programs, reducing the rate of virologic failure and preventing the spread of resistant HIV strains through targeted interventions.

## Introduction

The epidemic of HIV in Bangladesh is largely driven by people who inject drugs (PWID) and is mainly concentrated in the southern part of the capital city Dhaka.^6^ The HIV serological surveillance in 2016 showed that the overall prevalence of HIV among PWID in Dhaka City has significantly increased.^4^ However, the HIV prevalence declined among PWID in national surveillance conducted in 2020, with a fourfold increase among men who have sex with men (MSM).^7^ Over the past decade, the rising trend in new case detection has remained consistent,^8^ except for the years 2020 and 2021, which might be due to the COVID-19 pandemic's interruption to the healthcare system.^9^, ^10^, ^11^ In 2024, 1438 new cases were identified; the alarming issue is that the highest proportion of newly diagnosed HIV individuals was MSM (31%) whereas, the second highest was the general population (24%).^8^

The current harm reduction program faces challenges in tracking and screening the HIV-positive returnee migrants and their partners for enrollment into anti-retroviral therapy (ART) services. The main focus of the harm reduction program is to raise awareness among key populations (KPs) about safer sex using condoms and lubricants while providing needle-syringe and Opioid Substitution Therapy (OST) to PWID through drop-in-centers (DICs).^12^ These DICs also provide periodic HIV screening services to the KPs, and if HIV infection is detected, the participant is referred to ART centres for medications. Data from the Integrated Biological and Behavioural Survey (IBBS) conducted in 2016 indicated that 53% of PWID in Dhaka city shared needles and syringes in the last week and >30% of HIV-positive PWID actively engaged in sex; however, only <40% used condoms consistently.^13^ The latest IBBS conducted in 2020 indicated that only 41.8% of female sex workers (FSW) used condoms during sex with their clients within the last 4 weeks; 14.4% of MSM used condoms with commercial sex partners in the last 6 months, 41.3% of the transgender women (TGW) used condoms at last sex with men or TGW who paid for sex in past 6 months, and 15.6% of PWIDs used condoms during sex with a female commercial partner in the last 12 months.^7^ Despite the ongoing harm reduction program, this behaviour of KPs contributed to increasing HIV transmission.

On the other hand, previous studies indicated that HIV can be transmitted through unprotected sexual exposure from HIV-positive clients to their partner even having a low but detectable viral load (200 ≤ VL ≤ 1000 copies/mL).^14^^,^^15^ Unfortunately, Bangladesh has inadequate facilities for monitoring viral load among HIV-positive clients. However, the current national ART guideline recommends a VL test after 6 months of ART initiation and every 12 months thereafter or to measure CD4 count every 6 months if VL testing is unavailable.^16^ In addition, ART regimens have been suggested without checking the antiretroviral drug-resistant profile; while several mutations related to antiretroviral drug resistance were previously identified, even among ART naïve clients.^4^^,^^5^ This resistance could result in ART failure and be transmitted to new HIV-positive clients that gradually take the country into the HIV epidemic. This study aimed to identify drug resistance mutations among ART clients having a high VL.

## Methods

HIV-positive individuals who came to the ART centers to receive medication through the DICs's referral system were randomly approached and enrolled in the current study from March 2019 to November 2020. A total of 110 HIV-positive individuals were included who were from 20 different DICs located in 11 districts of Bangladesh. Participants' blood was collected in ethylenediamine tetra-acetic acid (EDTA) tubes at the DICs and was processed into plasma aliquots. Approximately 2 mLs plasma was shipped to the Virology Laboratory of icddr,b with the data collected during the enrollment, such as date of ART initiation and ART regimen name. A viral load (VL) test was conducted within 48 h of specimen collection using Xpert® HIV-1 Viral Load kits (Cepheid; USA). Participants with VL ≥ 1000 copies/mL are considered a virologic failure.^17^ One year after the first VL test, 20 participants were randomly selected (10 with VL < 1000, and 10 with >1000) to explore whether initial VL could predict virologic outcomes and treatment effectiveness over time.

Viral RNA was extracted from plasma samples having VL ≥ 1000 copies/mLs, using the QIAamp Viral RNA Mini Kit according to the manufacturer's protocol (QIAGEN, Leusden, Netherlands) and the *pol* gene was amplified by a nested RT-PCR as described elsewhere^4^ to cover the minimal regions for drug-resistance mutation (DRM) analysis.^18^ All PCR products were analyzed by agarose gel electrophoresis staining with GelRed™ (Biotium, Inc, CA) and purified using an ExoSAP-IT kit (Life Technology, Foster City, CA). Sequencing was performed in an Applied Biosystems® 3500 Genetic Analyzer using Big Dye® Terminator v3.1 Cycle Sequencing Ready Reaction kit (Life Technology). The chromatogram sequencing files were inspected using Chromas 2.23 (Technelysium, Queensland, Australia), and the consensus sequences were prepared using SeqMan II (DNASTAR, Madison, WI).

The time interval between ART initiation and the plasma specimen collection was analyzed using basic descriptive statistics to summarize the central tendency, either with chi-square or Kruskal–Wallis test to assess differences between groups. A time interval <1 month was considered as naïve to ART, while participants on ART for at least 6 months were included for analysing virologic failure.

HIV subtyping was done using REGA HIV-1 Automated Subtyping Tool V3^19^ and genotypic DRM profiles were obtained from the Stanford University HIV drug resistance database (HIVdb, Program Version 3.5.0, Algorithm Version 9.6, http://hivdb.stanford.edu/).^20^

At the same time, the quality of *pol* gene was also done using the HIVseq programs, and HIValg program was used for the comparison of drug resistance profile with: *i*. Rega Institute (KU Leuven; v10.0.0), and *ii*. Agence Nationale de Recherches sur le SIDA (ANRS; v33).

For phylogenetic analysis, all available Bangladeshi *pol* sequences were retrieved from HIV databases (www.hiv.lanl.gov) on June 23, 2024. The analysis was performed using MEGA-X (version 10.0.5). Using the Model Finder, we identified the General Time Reversible (GTR) model with a Gamma distribution (G) and invariable sites (+I) parameter (GTR + G + I) as the best substitution model and applied it with 1000 bootstrap replicates.

### Ethics statement

The current study was a supplement from the activity designed to observe VL among HIV-positive clients enrolled in the national harm reduction program and receiving support from a designated DICs. The activity was approved by icddr,b institutional review board. All participants provided written informed consent that the specimens could be used in future HIV research after removing the identifier.

### Role of the funding source

The study funders had no role in the study design, data collection, data analysis, interpretation, or writing of the report.

## Results

**Fig. 1:**
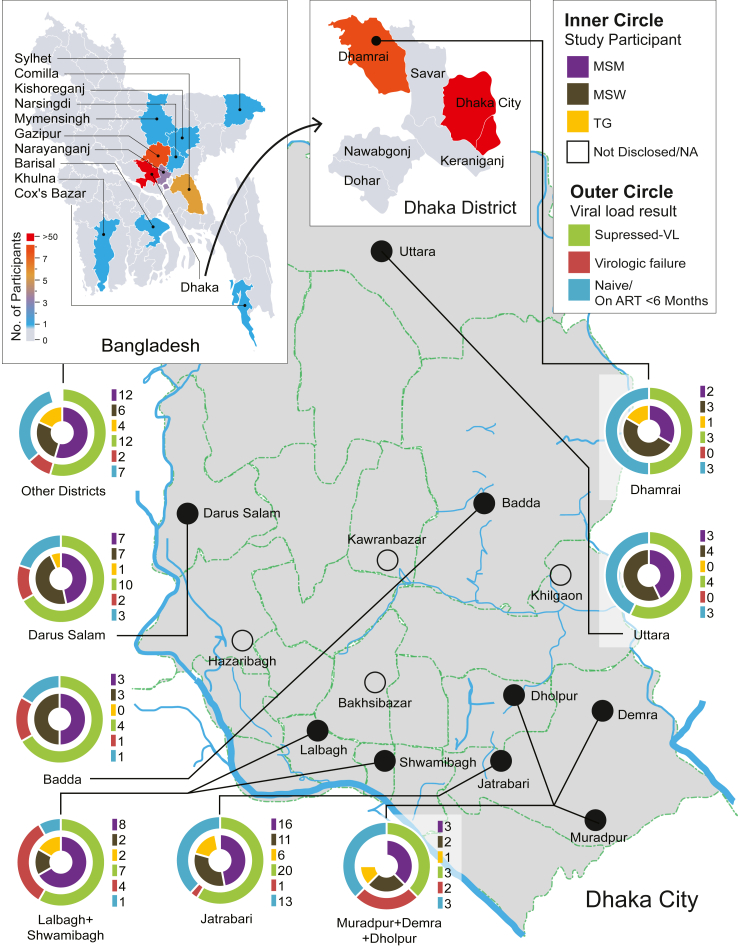
DIC-wise participant distribution and VL results.

Among 110 participants, 80% were from the capital city, Dhaka (Fig. 1). Considering the enrollment of risk groups, MSM, MSW and TGW were 54, 38, and 15 individuals, respectively; three participants did not disclose their identity. Overall, 28 years old was the median age (IQR: 24–35). The median age of MSM was 30 (IQR: 24.3–36), followed by TGW (27, IQR: 23.5–34) and MSW (26, IQR: 24.3–30.8); although, the differences between these three groups were statistically insignificant (*p* = 0.2).

The median time interval between ART initiation and the plasma specimen collection for VL was 11.5 months (IQR: 4.5–29.1; Min: 0 day; Max: 83 months). A total of 15 participants were naïve to ART at the time of specimen collection. Among participants who had been on ART for at least six months, 15.8% (12/76) experienced virologic failure (VL > 1000 copies/ml). In Dhaka city, the virologic failure rate was 16.4%, compared to 13.3% in participants from other districts (*p* = 0.4). Within Dhaka, participants from the southern part, which is considered as the epicentre for HIV in Bangladesh, had a virologic failure rate of 17.5% compared to 14.3% in the northern parts (*p* = 0.4) (Fig. 1).

**Fig. 2:**
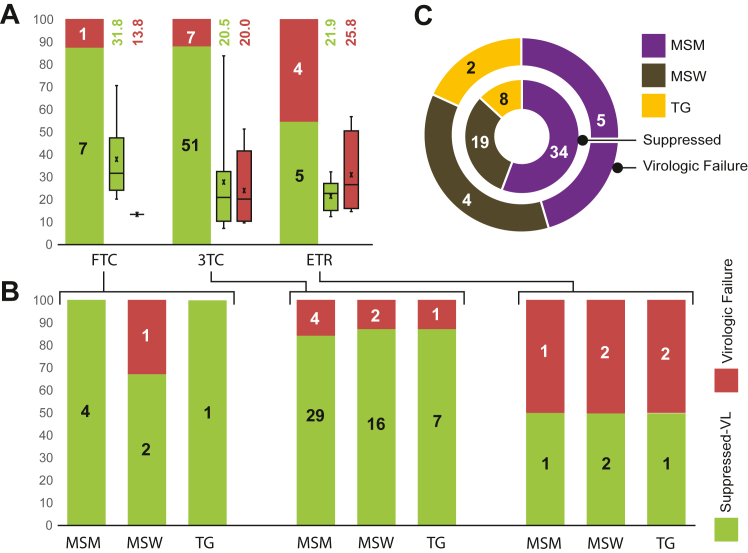
Treatment outcomes for different ART regimens on different KPs. **(A)** Treatment outcomes (VL results as barchart) with median time (as boxplot) for different ART regimens (name at the bottom). **(B)** Treatment outcomes among different populations for different regimens that shown in figure-A. **(C)** Overall treatment outcomes among different populations; where the inner circle represents viral suppression and the outer circle represents virologic failure.

ART was provided as a combination of three drugs where Efavirenz (EFV) and Tenofovir (TDF) were common. A third drug was added to the combinations, and the participants had been receiving three types of regimens with additional Lamivudine (3 TC), Etravirine (ETR), or Emtricitabine (FTC). A total of 78 (70.9%) participants were receiving 3 TC, 24 (21.8%) ETR, and 8 (7.3%) FTC. The median time for ART with EFV + TDF + ETR was lowest, 2.4 months (IQR: 0.8–29.13), followed by EFV + TDF+3 TC (median: 11.3 months, IQR: 6.3–29.9) and EFV + TDF + FTC (median: 28.4 months, IQR: 23.4–45.3) (Fig. 2A). It was observed that EFV + TDF + ETR was considered the first choice for initiating ART, with the highest number of naïve participants (n = 9) than EFV + TDF+3 TC (n = 6).

Participants receiving ART for at least six months, showed the highest virologic failure with EFV + TDF + ETR regimens (4/9, 44.4%), than EFV + TDF + FTC (1/8, 12.5%), and EFV + TDF+3 TC (7/59, 11.9%). However, there was no significant difference in ART duration between viral suppressed and virologic failure participants within each regimen. In addition, there was no difference in treatment outcomes among different populations for different regimens (Fig. 2B). However, when all regimens were analyzed together, TGW participants showed the highest virologic failure (20%, 2/10) compared to MSW (16.7%, 4/24) and MSM (12.8%, 5/39) (Fig. 2C).

Samples (n = 26) with high VL were subjected to *pol* gene sequencing in which, 16 (61.5%) were successfully sequenced for the protease (*prot*) and reverse transcriptase (*rt*) regions except one (only *rt* was successful). Overall, HIV-1 subtype C (43.8%, 7/16) was predominant, followed by A1 (25%, 4/16), CRF01_AE (25%, 4/16) and CRF02_AG (6.2%, 1/16). A similar genotypic distribution was observed among MSM and MSW participants.

**Fig. 3:**
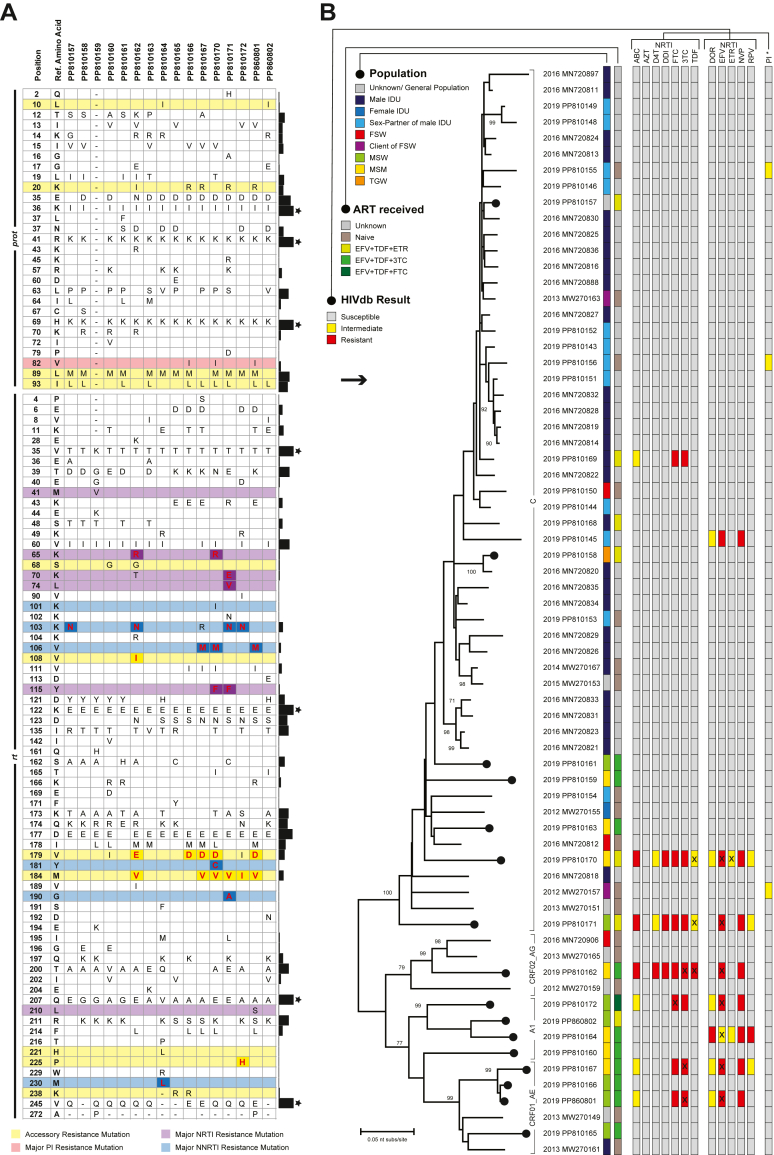
**(A)** The amino acid substitution in pol gene; **(B)** Maximum likelihood tree with resistant profile based on HIVdb databases for different KPs and different ART regimens.

The amino acid substitution chart of the sequenced sample identified several mutations, and some of them were at DRM sites (Fig. 3A). At *prot* DRM site, several mutations were observed, but none was associated with protease inhibitor (PIs) resistance. However, four mutations associated with nucleoside reverse transcriptase inhibitors (NRTI) resistance (K65R, K70E, L74V, and Y115F) and five associated with non-nucleoside reverse transcriptase inhibitors (NNRTI) resistance (K103N, V106M, Y181C, G190A, and M230L) were found in the study samples. These mutations might result in drug resistance (Supplementary Table S1) and virologic failure.

According to the phylogenetic tree, a large cluster of HIV genotype C was observed with a monophyletic branch of strains from intravenous drug user (IDU), their sex partner, and other KPs (Fig. 3B). In addition, strains from other KPs were distinctly clustered excluding IDU in other genogroups. Sequences from different populations and different ART were clustered together, indicating possible transmission within and between the KPs.

**Fig. 4:**
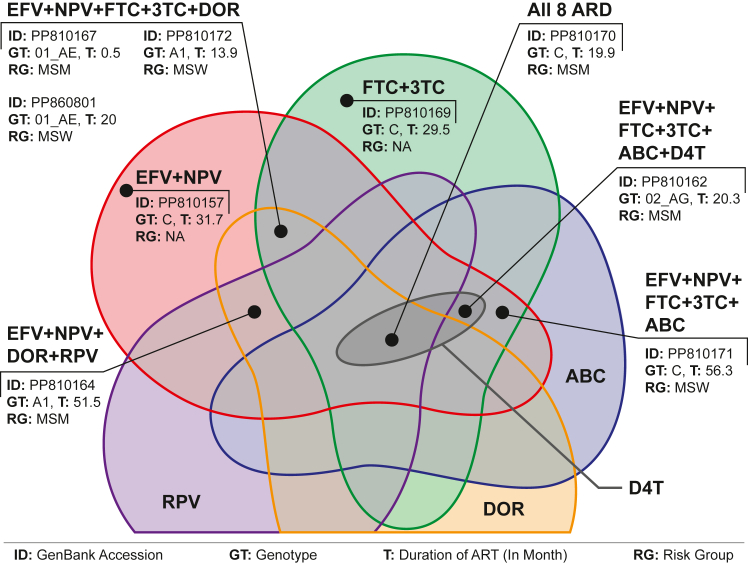
Venn-diagram showing samples' intersection for different drug resistance.

Drug resistance analysis using HIVdb algorithms showed that all samples were susceptible to PIs. Nine participants showed at least one NNRTI or NRTI drug resistance, and, among them one was ART-naïve but found resistance to EFV, NVP FTC and 3 TC. The median time interval between ART initiation was 24.5 months (IQR: 20–36; Min: 14; Max: 56) for the rest of the eight samples (Fig. 4). One participant showed resistance to all three regimens (EFV + TDF + ETR/FTC/3 TC) and two participants showed resistance to EFV + TDF + FTC/3 TC. Three samples showed resistance against both TDF and EFV, while an additional four were against EFV only. One participant who received FTC was resistant to it, and another six participants were FTC resistant, while three of them received 3 TC and another three ETR. Three participants who received 3 TC were resistant to it, and an additional four participants were 3 TC resistant while receiving ETR (n = 3) or FTC (n = 1). Only two samples showed intermediate resistance for ETR, one of them receiving it and another receiving 3 TC.

**Fig. 5:**
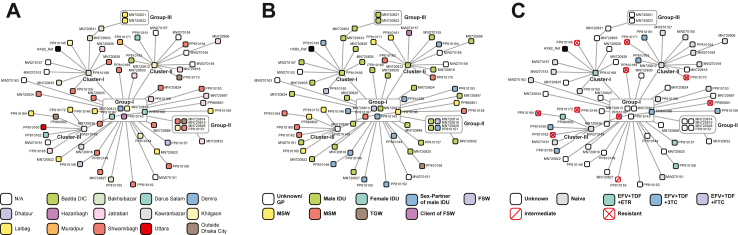
SNP-based transmission analysis **(A)** within different DIC, geographical locations; **(B)** between different KPs; and **(C)** for different ART regimens and outcomes.

Single Nucleotide Polymorphism (SNP) based transmission analysis revealed three clusters and three groups among the participants (Fig. 5). At the centre, Group-I contains 5 samples that played the crucial role as the primary transmitter. All three clusters, along with Group-II, came out from Group-I as branches. Other samples were also sporadically coming out from this group with one or two samples. Group-II contained 4 samples and Group-III, which was a branch of Cluster-I contained 2 samples.

The spatial distribution revealed a geographical distribution within the clusters and groups (Fig. 5A); only Cluster-I represents south Dhaka by having samples from Bakhsibazar, Lalbag, Shwamibagh, and Jatrabari. However, samples from Darus Salam, Demra, Hazaribagh, and Khilgaon areas were presented in Group-I and potentially acted as hotspots for infection spread. The risk group association highlights the potential concentration of transmission within these clusters and groups (Fig. 5B). Cluster-I, Group-II, and Group-III were mainly concentrated within IDU, their sex partners, and FSW. MSM and MSW were concentrated in Cluster-III; while Cluster-II and Group-I contained samples from diverse risk groups. Furthermore, treatment and resistance data were analyzed, revealing the homogenous distribution of the ART-resistant samples (Fig. 5C). In addition, samples that received different antiretroviral combinations clustered together.

A total of 20 participants receiving EFV + TDF+3 TC, were included in the follow-up; among them, 5 showed virologic failure. All ten participants with VL < 1000 copies/mL at the baseline showed viral clearance (<40 copies/mL) during follow-up. Among participants with VL < 1000 copies/mL at the baseline, 3 were naïve, of which 1 showed virologic failure (Table 1). Among recurrent 5 virologic failure participants, we successfully sequenced 4 samples, while three of them had major DRM.

**Table 1:** Follow-up data for participants with high VL at the baseline.

Lab ID/accession	Age (Year)	Group	Location	ART duration (Months)	VL (copies/mL)	Genotype	Resistance mutations		Follow-up VL (copies/mL)
							NRTI	NNRTI	
PP810160	36	MSM	Uttara	5.3	17,000	A1	S68G	–	<40
PP810161	23	MSW	Darus Salam	10.7	6260	C	–	–	32,500
PP810162	29	MSM	Darus Salam	20.3	61,900	CRF02_AG	K65R, S68G, K70T, M184V	K103N, V108I, V179E	56,600
PP810163	24	MSM	Badda	41.5	15,100	C	–	–	<40
PP810164	29	MSM	Lalbagh	51.5	21,800	A1	–	M230L	9510
VL-036	23	MSM	Demra	4.9	27,300	Failed			763,000
PP810165	25	MSW	Jatrabari	1.2	9550	CRF01_AE	–	–	216
PP810166	27	MSW	Darus Salam	0.6	3370	CRF01_AE	–	V179D	<40
VL-052	22	MSM	Jatrabari	0.6	1070	Failed			<40
PP810167	32	MSM	Darus Salam	0.5	313,000	CRF01_AE	M184V	V106M, V179D	231,000

## Discussion

The National ART program is currently aimed at achieving the Sustainable Development Goal (SDG) of ending AIDS as a public health threat by 2030. It is a signatory to the UN strategy of 95-95-95 by 2025^21^; however, the drug-resistance mutation (DRM) test is limited in the country.^4^^,^^5^ This study reported DIC-wise ART data and associated virologic responses from Bangladesh for the first time (Fig. 1). It highlights the presence of DRM within study participants who received ART and showed virologic failure, emphasizing the importance of integrating VL and DRM tests into the National AIDS/STD Programme (ASP) to achieve the SDG goal. Additionally, we provide evidence of DRM transmission within and between KPs.

Previous studies, mainly based on *gag* gene sequence, identified that genotype C was concentrated among IDU and mainly spread through using unsafe needles. In contrast, other genotypes spread through sexual routes and circulated among KPs, such as sex workers and migrant returnees.^4^^,^^5^^,^^22^, ^23^, ^24^ However, the active sexual life of IDU with transactional and non-transactional female sex partners^7^^,^^13^ increased spillover risk for genotype C to other KPs and vice-versa, introducing other genotypes among IDU. This dynamic was observed in our phylogenetic (Fig. 3B) and transmission (Fig. 4B) analyses. In the year 2024, 24% of the new HIV-positive cases were from the general population (GP),^8^ mainly those who did not disclose their behavioural status, creating a significant barrier to defining the transmission network (Cluster-II, Fig. 4B).

Currently, >7800 PLWH are on ART, and approximately 75% of them have received VL tests, of which 9% show virologic failure.^8^ However, this study observed comparatively high virologic failure (16%) among KPs who were taking ART for at least six months. It might be due to including KPs only, which is comparable with studies from other countries.^17^^,^^25^, ^26^, ^27^, ^28^ Additionally, the transmission network analysis identified clusters and groups with samples from different risk groups, treatments, and resistance patterns. This underscores the need for targeted interventions and tailored treatment strategies to address the distinct transmission patterns and resistance profiles within these populations, providing critical insights for public health strategies and policy formulation.

However, in Bangladesh, ASP still follows the “test and treat strategy” to initiate ART, where TDF+3TC/FTC + DTG or TDF+3TC/FTC + EFV are considered as first-line regimens, while AZT+3 TC + EFV or TDF/TAF+3TC/FTC + PI/r are considered during special situations.^16^ Our analysis identified several DRMs within the *pol* gene and potential drug resistance (Fig. 3), which was further confirmed by follow-up VL tests (Table 1). Therefore, it is high time to move forward with updating treatment protocols.

This cross-sectional study has some limitations, and the results should be considered with caution. First, the small sample size may not fully represent the broader population of PLWH in Bangladesh. Additionally, the majority of participants were from the capital city, Dhaka, which may not represent the diverse HIV epidemic across different regions of Bangladesh. This may also lead to a selection bias where 21.8% of the participants were receiving TDF/EFV/ETR regimen. This regimen is not a standard regimen and is not commonly listed as an alternative,^16^ but it has been used in certain cases, particularly when drug availability limits other options. Second, we were unable to sequence 38.5% of samples with high viral load, possibly due to mutations at the primer binding sites or technical limitations in the sequencing process. Third, the DRM and resistance analyses were based on partial *pol* sequences rather than full-gene sequencing. These two limitations could result in an underestimation of resistance mutations and may miss mutations outside the targeted regions, potentially affecting the accuracy and completeness of the resistance profiles. Fourth, this study did not extensively collect or analyze data on participants' adherence to ART or other behavioural factors that might influence virologic outcomes.

Despite these limitations, the study provides valuable insights into HIV treatment and drug resistance in Bangladesh. CD4 is no longer considered a reliable substitute for ART failure detection,^29^ leading to the recommendation of removing the routine CD4 testing every 6 months from the national ART guideline and focusing on strengthening routine VL monitoring as per WHO recommendations, ensuring VL testing is prioritized for treatment monitoring and decision-making. Although pre-ART resistance screening is not necessary for the TDF+3TC/FTC + DTG first-line regimen, we strongly recommend integrating a DRM monitoring system alongside VL testing, particularly in resource-limited countries like Bangladesh, where the availability of this regimen may be inconsistent or interrupted. This is a critical requirement for improving the effectiveness of the ASP. Although national ART guidelines emphasise routine VL and DRM testing, this study recommends immediate policy reform for the current ART management framework by mandating DRM testing before initiating ART and regular VL testing thereafter. Strengthening health systems to support the scale-up of VL and DRM testing may be necessary. Such policy shifts are essential to advancing toward the Sustainable Development Goal of ending AIDS as a public health threat by 2030.

Finally, our evidence suggests that KPs are at a higher risk of virologic failure in Bangladesh. Therefore, public health strategies should include tailored outreach and education programs aimed at preventing the transmission of resistant HIVwithin and between KPs. Furthermore, without integrating DRM testing into ART programs, achieving the UNAIDS 95-95-95 targets will remain challenging, potentially hindering progress in controlling the HIV epidemic. Finally, this study highlights the critical need for enhanced monitoring, targeted interventions, and policy reforms in ART programs in Bangladesh. In addition, longitudinal studies with large sample sizes may be required to track treatment outcomes over time. These actions are essential for improving patient outcomes, preventing the spread of drug-resistant HIV, and achieving global health goals related to HIV/AIDS.

## Contributors

SR, GS, SIK, and MR designed this study. MSS supervised the laboratory work, conducted by MAH, MF, RH. SR and MMH planned and conducted all analyses including bioinformatics and statistics. GS supervised the field team and monitored the enrolment for data and sample collection. GS and SIK directly accessed and verified the field data, while MSS accessed and verified the laboratory data. SR wrote the manuscript. All authors reviewed and approved the final version of the manuscript, had full access to all data in the trial, and had final responsibility for the decision to submit for publication.

## Data sharing statement

All sequence generated in this study was submitted to the GenBank database under accession no: PP810143– PP810172, PP860801-PP860802. The data supporting the findings of this study are available with this article as a Supplementary file. Additional data can be obtained from the corresponding author upon reasonable request.

## Declaration of interests

MR is part of the IRB, icddrb, however, he was not a part of the IRB or the review committee (approved in 2019), for the current study.

The authors declare that they have no known competing financial interests or personal relationships that could have appeared to influence the work reported in this paper.
