# Supplementary material for: Drug resistance mutations among people living with HIV and ART failure in Bangladesh: a cross-sectional study
Source: Lancet Reg Health Southeast Asia. 2025 Jul 4;40:100629. doi: 10.1016/j.lansea.2025.100629 (PMC12272934; doi:10.1016/j.lansea.2025.100629)
Supplement: Supplementary Table [file mmc1.pdf]

Table S1: Drug resistance result based on different databases and presence of mutation on *rt* region of *pol* gene.
Mutation on *prot* region was not presented as no resistance was observed for the protease inhibitor drug. Drugs with green fill colour represent susceptible (S), blue for possible/ intermediate resistance (I) and red for resistance (R) strain. DRM on *rt* were presented in red text.

GenBank Accession (Risk Group)	Genotype	Duration of ART (In Months)	Database	DOR	EFV	ETR	NVP	RPV	ABC	AZT	D4T	DDI	FTC	3TC	TDF	Mutation in *rt*
				NNRTI					NRTI							
PP810157 (N/A)	C	31.7	HIVDB	S	R	S	R	S	S	S	S	S	S	S	S	V35T, E36A, T39D, S48T, V60I, **K103N,** D121Y, K122E, I135R, S162A, K173T, Q174K, D177E, T200A, Q207E, V245Q
			ANRS	S	R	S	R	S	S	S			S	S	S	
			REGA		R	S	R	S	S	S	S	S	S	S	S	
PP810158 (TGW)	C	14.1	HIVDB	S	S	S	S	S	S	S	S	S	S	S	S	V35T, T39D, S48T, V60I, D121Y, K122E, I135T, S162A, K173A, Q174K, D177E, G196E, Q197K, T200A, Q207G, R211K
			ANRS	S	S	S	S	S	S	S			S	S	S	
			REGA		S	S	S	S	S	S	S	S	S	S	S	
PP810159 (MSM)	C	7.5	HIVDB	S	S	S	S	S	S	S	S	S	S	S	S	V35K, T39G, E40G, M41V, E44K, S48T, V60I, D121Y, K122E, I135T, Q161H, S162A, K173A, Q174R, D177E, I178L, E194K, Q197K, T200A, Q207G, R211K, V245Q, A272P, K277G, V292I, I293V, P313T, I326V, Q334N, G335D
			ANRS	S	S	S	S	S	S	S			S	S	S	
			REGA		S	S	S	S	S	S	S	S	S	S	S	
PP810160 (MSM)	A1	5.3	HIVDB	S	S	S	S	S	S	S	S	S	S	S	S	K11T, V35T, T39E, V60I, **S68G**, D121Y, K122E, I135T, I142V, K166R, E169D, K173A, Q174R, D177E, I178L, V179I, G196E, T200V, I202V, Q207A, R211K, V245Q
			ANRS	S	S	S	S	S	S	S			S	S	S	
			REGA		S	S	S	S	S	S	S	S	S	S	S	
PP810161 (MSW)	C	10.7	HIVDB	S	S	S	S	S	S	S	S	S	S	S	S	V35R, T39D, S48T, V60I, D121Y, K122E, S162H, K166R, K173T, Q174E, T200A, Q207G, R211K, V245Q
			ANRS	S	S	S	S	S	S	S			S	S	S	
			REGA		S	S	S	S	S	S	S	S	S	S	S	
PP810162 (MSM)	02_AG	20.3	HIVDB	S	R	I	R	I	R	S	R	R	R	R	R	E28K, V35T, V60I, **K65R, S68G**, **K70T, K103N,** K104R, **V108I,** K122E, D123N, I135T, S162A, K173A, Q174R, D177E, I178M, **V179E, M184V,** V189I, T200A, Q207E, F214L, V245Q
			ANRS	S	R	S	R	S	R	S			R	R	R	
			REGA		R	S	R	S	R	S	R	R	R	R	I	
PP810163 (MSM)	C	41.5	HIVDB	S	S	S	S	S	S	S	S	S	S	S	S	V8I, V35T, E36A, T39D, S48T, V60I, K122E, I135V, D177E, I178M, T200E, E204K, Q207A, V245Q
			ANRS	S	S	S	S	S	S	S			S	S	S	
			REGA		S	S	S	S	S	S	S	S	S	S	S	
PP810164 (MSM)	A1	51.5	HIVDB	R	I	I	R	R	S	S	S	S	S	S	S	K11E, V35T, K49R, V60I, D121H, K122E, D123S, K173T, Q174K, D177E, S191F, I195M, Q197K, T200Q, Q207V, R211K, T216P, H221L, W229R, **M230L**
			ANRS	R	R	S	R	R	S	S			S	S	S	
			REGA		R	I	R	I	S	S	S	S	S	S	S	

GenBank Accession (Risk Group)	Genotype	Duration of ART (In Months)	Database	DOR	EFV	ETR	NVP	RPV	ABC	AZT	D4T	DDI	FTC	3TC	TDF	Mutation in *rt*
				NNRTI					NRTI							
PP810165 (MSW)	01_AE	1.2	HIVDB	S	S	S	S	S	S	S	S	S	S	S	S	E6D, V35T, T39K, K43E, K122E, D123S, I135R, S162C, F171Y, Q174K, D177E, I202V, Q207A, R211S, K238R
			ANRS	S	S	S	S	S	S	S			S	S	S	
			REGA		S	S	S	S	S	S	S	S	S	S	S	
PP810166 (MSW)	01_AE	0.6	HIVDB	S	I	I	I	I	S	S	S	S	S	S	S	E6D, K11T, V35T, T39K, K43E, V111I, K122E, D123S, D177E, I178M, **V179D,** Q207A, R211S, F214L, K238R, V245E, T286A, E291D, V292I, I293V, P294T, E312T, V317T, I326V, I329V, G335D, M357R, G359S, K366R, A371V, A376T, T377M, V381I, T386I, K390R, A400T, T403M, W406R
			ANRS	S	S	S	S	I	S	S			S	S	S	
			REGA		S	S	S	S	S	S	S	S	S	S	S	
PP810167 (MSM)	01_AE	0.5	HIVDB	S	R	I	R	I	I	S	S	S	R	R	S	P4S, E6D, K11T, V35T, T39K, K43E, V60I, **K103R, V106M**, V111I, K122E, D123N, D177E, I178M, **V179D, M184V,** Q207A, R211S, F214L, V245E, D250S
			ANRS	R	R	S	R	I	I	S			R	R	S	
			REGA		R	S	R	S	S	S	S	S	R	R	S	
PP810170 (MSM)	C	19.9	HIVDB	R	R	I	R	I	R	S	S	R	R	R	R	V35T, T39N, V60I, **K65R**, K101I, **V106M,** V111I, **Y115F,** K122E, D123N, I135T, T165I, K173T, D177E, I178L, **V179D, Y181C, M184V,** T200A, Q207E, R211K, F214L, V245Q
			ANRS	R	R	R	R	R	R	S			R	R	R	
			REGA		R	I	R	I	R	S	R	R	R	R	I	
PP810171 (MSW)	C	56.3	HIVDB	S	R	I	R	I	R	S	S	R	R	R	I	V35T, T39E, K43R, **K70E, L74V,** K102N, **K103N, Y115F,** K122E, D123S, S162C, K173A, D177E, **M184V, G 190A,** I195L, Q197K, T200E, Q207E, V245Q
			ANRS	S	R	S	R	S	R	S			R	R	R	
			REGA		R	S	R	S	R	S	S	R	R	R	I	
PP810172 (MSW)	A1	13.9	HIVDB	S	R	S	R	S	I	S	S	S	R	R	S	E6D, V35T, E40D, K49R, V60I, V90I, **K103N,** K122E, D123N, I135T, K173S, Q174N, D177E, V179I, **M184I,** T200A, Q207A, R211K, V245Q, **P225H,** E248D
			ANRS	R	R	I	R	S	I	S			R	R	S	
			REGA		R	S	R	S	S	S	S	S	R	R	S	
PP860801 (MSW)	01_AE	20.0	HIVDB	S	R	S	R	S	I	S	S	S	R	R	S	E6D, K11T, V35T, T39K, K43E, **V106M**, V111I, K122E, D123S, K166R, D177E, I178M,**V179D**, **M184V,** Q207A, L210S, R211S, F214L, V245E, A272P, K275R, V276VI
			ANRS	R	R	S	R	S	I	S			R	R	S	
			REGA		R	S	R	S	S	S	S	S	R	R	S	
PP860802 (MSW)	A1	0.2	HIVDB	S	S	S	S	S	S	S	S	S	S	S	S	V8I, K11E, V35T, V60I, D113E, D121H, K122E, D123S, I135T, T165I, K173A, Q174K, D177E, D192N, Q197K, T200A, I202V, Q207A, R211K
			ANRS	S	S	S	S	S	S	S			S	S	S	
			REGA		S	S	S	S	S	S	S	S	S	S	S	
PP810168 (N/A)	C	63.6	HIVDB	S	S	S	S	S	S	I	S	S	S	S	S	V35T, E36A, T39D, S48T, V60I, T69I, D121Y, K122E, I135R, S162T, K166R, K173A, D177E, T200A, **K219N**
			ANRS	S	S	S	S	S	S	S			S	S	S	
			REGA		S	S	S	S	S	S	S	S	S	S	S	
PP810169 (N/A)	C	29.5	HIVDB	S	S	S	S	S	I	S	S	S	R	R	S	V35T, E36A, T39D, S48T, V60I, T69I, D121Y, K122E, I135K, S162A, K173A, Q174K, D177E, **M184V,** T200A, V245Q
			ANRS	S	S	S	S	S	I	S			R	R	S	
			REGA		S	S	S	S	S	S	S	S	R	R	S	
